# Hepatobiliary cystadenoma exhibiting morphologic changes from simple hepatic cyst shown by 11-year follow up imagings

**DOI:** 10.1186/1477-7819-6-129

**Published:** 2008-12-11

**Authors:** Naoto Fukunaga, Masashi Ishikawa, Hisashi Ishikura, Toshihiro Ichimori, Suguru Kimura, Akihiro Sakata, Koichi Sato, Jyunichi Nagata, Yoshiyuki Fujii

**Affiliations:** 1 Department of Surgery, Tokushima Red Cross Hospital, Komatsushima-City, Tokushima Prefecture, Japan; 2 Department of Gastroenteology, Tokushima Red Cross Hospital, Komatsushima-City, Tokushima Prefecture, Japan; 3 Department of Pathology, Tokushima Red Cross Hospital, Komatsushima-City, Tokushima Prefecture, Japan

## Abstract

**Background:**

A long-term follow up case of hepatobiliary cystadenoma originating from simple hepatic cyst is rare.

**Case presentation:**

We report a case of progressive morphologic changes from simple hepatic cyst to hepatobiliary cystadenoma by 11 – year follow up imaging. A 25-year-old man visited our hospital in 1993 for a simple hepatic cyst. The cyst was located in the left lobe of the liver, was 6 cm in diameter, and did not exhibit calcification, septa or papillary projections. No surgical treatment was performed, although the cyst was observed to gradually enlarge upon subsequent examination. The patient was admitted to our hospital in 2004 due to epigastralgia. Re-examination of the simple hepatic cyst revealed mounting calcification and septa. Abdominal CT on admission revealed a hepatic cyst over 10 cm in diameter and a high-density area within the thickened wall. MRI revealed a mass of low intensity and partly high intensity on a T1-weighted image. Abdominal angiography revealed hypovascular tumor. The serum levels of AST and ALT were elevated slightly, but tumor markers were within normal ranges. Left lobectomy of the liver was performed with diagnosis of hepatobiliary cystadenoma or hepatobiliary cystadenocarcinoma. The resected specimen had a solid component with papillary projections and the cyst was filled with liquid-like muddy bile. Histologically, the inner layer of the cyst was lined with columnar epithelium showing mild grade dysplasia. On the basis of these findings, hepatobiliary cystadenoma was diagnosed.

**Conclusion:**

We believe this case provides evidence of a simple hepatic cyst gradually changing into hepatobiliary cystadenoma.

## Background

Hepatobiliary cystadenoma is a rare benign tumor arising from the liver, or less frequently from the extrahepatic biliary tree. Edmondson et al [1]. reported the definition of the hepatobiliary cystadenoma for the first time in 1958. It accounts for 4.6% of intrahepatic cysts of bile duct origin and the most frequently occurs in middle-aged women[2]. In 1985, Wheeler and Edmondson[3] described distinct criteria for hepatobiliary cystadenoma based on the presence or absence of mesenchymal stroma. Cystadenoma lacking mesenchymal stroma predominantly occurred in males while cystadenoma with mesenchymal stroma is composed of intermediate stroma components and is most prevalent in females. It is characterized by multilocular cyst with a solid component, septa, papillary projections, or mural nodules[4]. Although the clinical and pathological findings of hepatobiliary cystadenoma and cystadenocarcinoma have been well-described, it cannot be distinguished from one another by imaging findings including computed tomography (CT), magnetic resonance imagings (MRI) and ultrasound (US). Moreover, hepatobiliary cystadenoma and simple hepatic cysts can change into hepatobiliary cystadenocarcinoma with time[2,5]. Although histopathological differentiation between hepatobiliary cystadenoma and cystadenocarcinoma is indisputable, it is unknown whether hepatobiliary cystadenocarcinomas arise de-novo come or whether they arise from hepatobiliary cystadenomas. A long-term follow up study of hepatobiliary cystadenoma may contribute to the clarification of this sequence. Herein, we report a case of hepatobiliary cystadenoma with morphologic changes from simple hepatic cyst by 11-year follow up imaging.

## Case presentation

**Figure 1:**
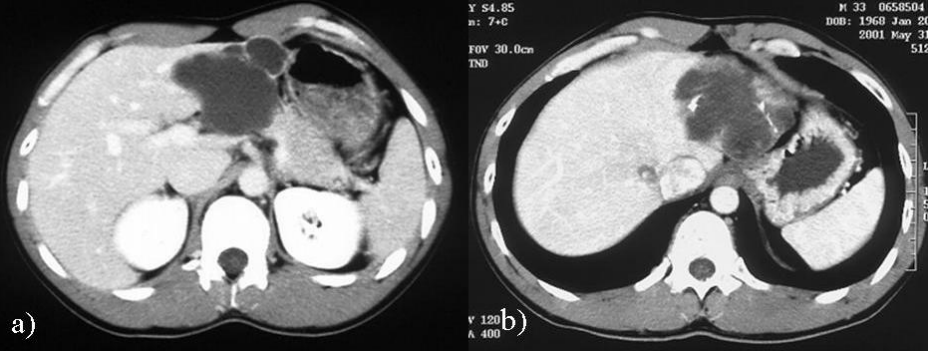
**Abdominal CT findings**. a) in 1996, showing the unilocular cyst 6 cm in diameter without calcification, septa and papillary projections. No contrast enhancement was seen. b) in 2001, showing the same diameter with calcification and septa.

**Figure 2:**
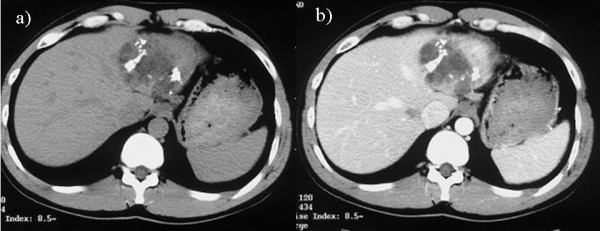
**Abdominal CT findings in 2004**. a) showing the unilocular cyst over 10 cm in diameter, increasing eruplioid calcification, septa and thickness of the wall within the cyst. b) the contrast was seen a little at the left side of the cyst.

**Figure 3:**
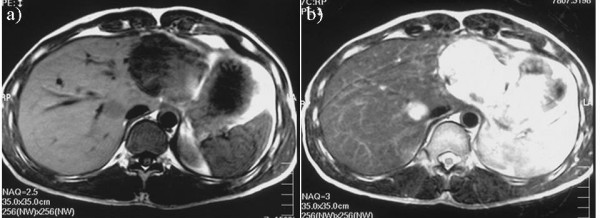
**Abdominal MRI in 2004, showing the unilocular cyst 10 cm in diameter**. a) low intensity, partly high intensity on T1-weighted image, b) high intensity on T2-weighted image were seen.

**Figure 4:**
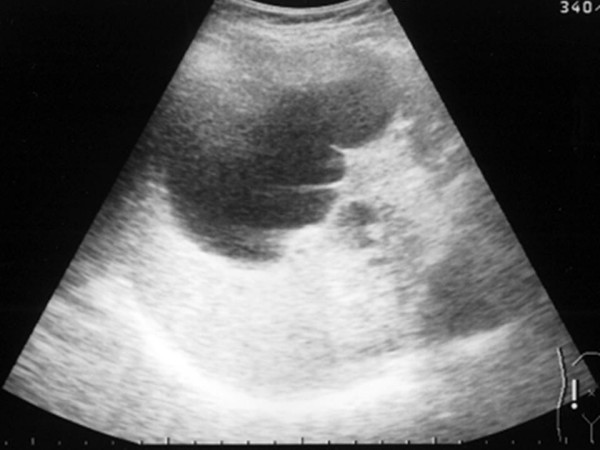
**Abdominal US in 2004, showing the unilocular cyst over 10 cm in diameter**. Partially, the septa within the cyst were seen.

**Figure 5:**
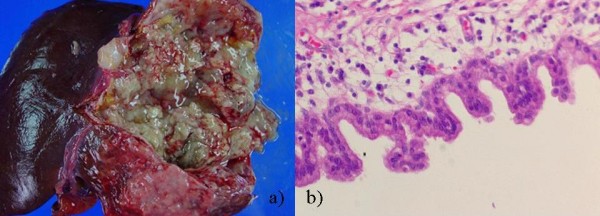
**Cut surface and pathological findings in 2004**. a) Cut surface, showing elastic white-colored scar and yellowish papillary nodule. b) Pathological finding, showing the inner layer of the cyst was lined with a columnar epithelium exhibiting mild grade dysplasia, partially with a papillary epithelium.

A simple hepatic cyst was detected in the left lobe of the liver of a 25-year-old man in 1993 (Sadly, there was no imaging.). The patient was followed in our hospital, and no surgical treatment was performed although the cyst showed gradual enlargement. The patient was admitted to our hospital due to epigastralgia and for re-examination of simple hepatic cyst in 2004. In 1996 the cyst was unilocular, 6 cm in diameter without calcification, septa or papillary projections as observed by CT (Fig. 1a). In 2001 the cyst remained the same diameter but exhibited calcification and septa (Fig. 1b). An abdominal CT performed on admission in 2004 showed that the unilocular cyst had grown to over 10 cm in diameter with increasing mounting calcification, septa and thickening of the wall within the cyst (Fig. 2a, b). MRI revealed a partly low intensity, partly high intensity T1-weighted image, and high intensity T2-weighted image (Fig. 3a, b). US revealed a unilocular cyst over 10 cm in diameter and partial septa within the cyst (Fig. 4). Abdominal angiography showed the tumor to be hypovascular and stretching of left hepatic artery. Endoscopic retrograde cholangiopancreatography (ERCP) revealed compression of the bile duct and no communication between the cyst and the bile duct was shown. The serum level of aspartame aminotransferase (AST) and almandine aminotransferase (ALT) were slightly elevated but tumor markers such as CEA and CA 19-9 were within the normal range. The cystic lesion was suspected of being a mucin-producing liver tumor, such as hepatobiliary cystadenoma or cystadenocarcinoma. Despite of these findings, we could not rule out the malignancy clearly. Therefore, in November 2004, left lobectomy of the liver with cholecystectomy was performed. Macroscopically, a resected specimen was a unilocular tumor filled with mucus. The cut surface of the tumor exhibited an elastic white-colored scar and yellowish papillary nodule (Fig. 5a). The unilocular cyst had a solid component with papillary projections, septa and calcification and was filled with liquid-like muddy bile. Histopathological examination revealed that the inner layer of the cyst was lined columnar epithelium exhibiting mild grade dysplasia and partially lined with papillary epithelium (Fig. 5b). Dense mesenchymal stroma was not detected. On the basis of these findings, hepatobiliary cystadenoma was diagnosed. The postoperative course was uneventful and recurrence of the lesion has not been observed. We believe this case provides evidence of a simple hepatic cyst changing into hepatobiliary cystadenoma over a 10-year period.

## Discussion

Hepatobiliary cystadenoma is a rare benign tumor arising from the epithelium [2]. Hepatobiliary cystadenoma is reported to be defined as multilocular cystic tumors lined with columnar epithelium and containing dense cellular stroma.

In general, hepatobiliary cystadenoma was described as multilobular cyst with smooth surfaces and the vasculature externally [3]. The tumor tissue was also described such as white, grey – white, pink and so on. The internal surface of the tumor was generally smooth with occasional trabeculations, sessile or polypoid cysts. Hepatobiliary cystadenoma containted clear or turbid fluid described as mucinous or gelatinous, which was quantified from 700 to 4200 ml.

As to microscopic features in details, hepatobiliary cystadenoma consisted of following three layers; 1) the epithelial layer of mucin producing columnar to cuboidal cells lining within the cysts; 2) the layer, less than 3 mm in thickness of undifferentiated mesenchmal cells; 3) the outer layer, which was the dense layer with collagenous connective tissue.

Tumor size varies from 8 to 20 cm, with a mean of 13 cm [6]. Symptoms are various, including an upper abdominal mass, epigastralgia and abdominal pain. Asymptomatic lesions may be discovered incidentally during radiological or surgical procedures for unrelated conditions. Jaundice due to compression of the bile duct [6] and ascites due to compression of the vena cava and hepatic vein are rare. Laboratory examination is normal in most patients, although some exhibit mild elevated serum liver enzymes due to compression of the cystic mass. Tumor markers are also not unusually elevated, although Lee et al [7], revealed high serum CA 19-9 and the presence of CA 19-9 and CEA in the epithelial component of hepatobiliary cystadenoma by immunohistochemical analysis. Our case exhibited frequent symptoms and was diagnosed with a simple hepatic cyst by US and CT in 1993, although the cystic mass showed enlargement with internal septa and papillary projections. The characteristic CT findings of hepatobiliary cystadenoma are low-density well-subscribed masses with internal septa, mural nodules and papillary projections [4,6]. Contrast enhancement is often seen along the internal septa and wall. The US findings are also ovoid, cystic masses with multiple echogenic septa and papillary projections along the wall or septa [4,6,8]. Takayasu et al [8], have reported that US and CT are useful tools to clarify internal structure of the tumors and that make it easy to determine the preoperative diagnosis, but Matsumoto et al [4], reported that with regard to the internal structure, US was superior to CT in demonstration of internal morphology. Our case showed enlargement of the cyst with internal septa and papillary projections and emerging dense calcification along the wall and internal septa, being atypical of hepatobiliary cystadenoma. In particular, the presence of calcification along the wall or septa was reported to indicate hepatobiliary cystadenocarcinoma[4]. MRI is useful to evaluate the contents of the cysts such as mucin or hemorrhage[4]. ERCP is often used to show communication between hepatobiliary cystadenoma and intrahepatic duct. In some cases, a communication between the biliary tract and the tumor are shown by ERCP or intraoperative cholangiography. Angiographic findings are not diagnostic, but stretching of the hepatic arteries and irregular calibers of the peripheral arteries in the arterial phase and stains in the parenchymal phase lead to the suspicion of malignancy[9]. Hepatobiliary cystadenoma should be suspected by neovascularity with a thin rim of contrast material accumulating within the cysts[8]. Furthermore, in general, hemorrhagic internal fluid is suggestive of hepatobiliary cystadenocarcinoma, whereas mixed or mucinous fluid is suggestive of hepatobiliary cystadenoma. Certainly, imaging findings characteristic of hepatobiliary cystadenoma are recognized, but the differential diagnosis between hepatobiliary cystadenoma and hepatobiliary cystadenocarcinoma on the basis of imaging findings alone has not been established [4]. Some hepatobiliary cystadenoma and simple hepatic cysts are reported to show malignant transformation into hepatobiliary cystadenocarcinoma after a number of years[2,5]. As mentioned above, hepatobiliary cystadenoma was classified based on the presence or absence of mesenchymal stroma. Cystadenoma with mesenchymal stroma, which occured in females had the malignant transformation into cystadenocarcinoma with stromal invasion. There has been the possible histogenesis, respectively. Devaney et al[10], divided hepatobiliary cystadenocarcinoma into two groups; 1) that arising from preexisting cystadenoma with mesenchymal stroma, which predominantly occurred in females with an indolent clinical course; 2) that not associated with preexisting cystadenoma mesenchymal stroma, which occurred in males having an extremely aggressive clinical course. On the other hand, hepatobiliary cystadenoma with mesenchymal stroma may arise from ectopic ovary incorporated into the liver or ectopic rests of primitive tissue such as embryonic gallbladder and bile ducts, while that without mesenchymal stroma may originate from bile buct epithelium as reactions induced by various stimuli[3]. Ishak et al[2], reported the theories of origin of hepatic cyst. We speculated our case without mesenchymal stroma was originated from simple hepatic cyst as reactions by some stimuli, which were not unknown. Akiyoshi et al[11], reported a case of hepatobiliary cystadenocarcinoma with progression from a benign cystic lesion over 12 years. In their case, a small cyst grew by only 3 cm in diameter over 12 years and become malignant. We considered that malignant formation was not related to the rate of increase in the size of the cyst and took the malignancy based on the presence of calcification, malignant potential of benign cysts reported and the recurrence of hepatobiliary cystadenoma despite the presence of mesenchymal stroma into consideration. In our case, the progressive morphologic changes including enlargement of the cyst from 6 cm to 10 cm, septa, increasing calcification and thickness of the wall was recognized. Therefore, we performed the complete surgical resection. Kosuge et al[12], reported that the postoperative recurrence in patients who underwent radical resection for hepatobiliary cystadenocarcinoma was much less than that of patients with other hepatic malignancies. In our case, the surgical margin was negative and long-term survival would be expected. The benefit of chemotherapy has not still established in patients with palliative resection or distant metastasis.

## Conclusion

We report a case of hepatobiliary cystadenoma with morphologic changes from simple hepatic cyst shown by 11-year follow up imagings. Fortunately, complete radical resection was performed and no recurrence has been observed to date. Complete resection is mandatory surgical procedure, when hepatobiliary cystadenoma showing atypical imaging findings is suspected, or the malignancy cannot be denied.

## Consent

Written informed consent was obtained from the for publication of this case report and any accompany images. A copy of written consent is available for review by the Editor-Chief of this journal.

## Competing interests

The authors declare that they have no competing interests.

## Authors' contributions

NF wrote this manuscript and revised it. MI performed the operation. He conceptualize and recommended me to write this case and advised me to revise it. HI performed the operation and conceptualize and recommended me to write this case. TI performed the operation and conceptualize and recommended me to write this case. SK performed the operation and conceptualize and recommended me to write this case. AS performed the operation and conceptualize and recommended me to write this case. KS participated in the design of this case. JN participated in the design. YF made a diagnosis of this case histologically and participated in the design. All authors read and approved the final manuscript.
